# Supplementary material for: COVID-19 genetic risk variants are associated with expression of multiple genes in diverse immune cell types
Source: Nat Commun. 2021 Nov 19;12:6760. doi: 10.1038/s41467-021-26888-3 (PMC8604964; doi:10.1038/s41467-021-26888-3)
Supplement: Supplementary file 1 — Supplementary Information [file 41467_2021_26888_MOESM1_ESM.pdf]

Supplementary Information

COVID-19 genetic risk variants are associated with expression of multiple genes in diverse immune cell types.

Benjamin J. Schmiedel[1]†, Job Rocha[1,2]†, Cristian Gonzalez-Colin[1,2]†, Sourya Bhattacharyya[1]†, Ariel Madrigal[1], Christian H. Ottensmeier[1,3], Ferhat Ay[1,4]‡, Vivek Chandra[1]‡, Pandurangan Vijayanand[1,3]‡*

[1] La Jolla Institute for Immunology, La Jolla, CA, USA.

[2] Center for Genomic Sciences, National Autonomous University of Mexico, Cuernavaca, Morelos, Mexico.

[3] Liverpool Head and Neck Centre, Institute of Systems, Molecular and Integrative Biology, University of Liverpool, United Kingdom.

[4] Department of Pediatrics, University of California San Diego, La Jolla, CA, USA.

[5] Department of Medicine, University of California San Diego, La Jolla, CA, USA.

† These authors contributed equally.

‡ These authors jointly supervised this work.

* Corresponding author. Email: vijay@lji.org

Supplementary Figure 1

a

	COVID-19 GWAS phenotype description (https://www.covid19hg.org)	COVID-19 GWAS meta-analysis	GWAS meta-analysis (release 5 from January 18, 2021)	Total cases	Total controls	COVID-19 GWAS lead SNPs
A2	Very severe respiratory confirmed COVID-19 vs. population	**Critical illness**	A2_ALL_leave_23andme	5,582	709,010	698
			A2_ALL_leave_UKBB_23andme	5,870	1,155,203	655
			A2_ALL_eur_leave_23andme	5,101	1,383,241	654
			A2_ALL_eur_leave_ukbb_23andme	4,792	1,054,664	535
B1	Hospitalized COVID-19 vs. not hospitalized COVID-19	**Hospitalization**	B1_ALL_leave_23andme	5,773	15,497	190
			B1_ALL_leave_UKBB_23andme	3,961	10,538	128
			B1_ALL_eur_leave_23andme	4,829	11,816	38
			B1_ALL_eur_leave_ukbb_23andme	3,159	7,206	0
B2	Hospitalized COVID-19 vs. population	**Hospitalization**	B2_ALL_leave_23andme	12,888	1,295,966	1,765
			B2_ALL_leave_UKBB_23andme	11,829	1,725,210	756
			B2_ALL_eur_leave_23andme	9,986	1,877,672	714
			B2_ALL_eur_leave_ukbb_23andme	8,316	1,549,095	752
C2	COVID-19 infection vs. population	**Infection risk**	C2_ALL_leave_23andme	36,590	1,668,938	499
			C2_ALL_leave_UKBB_23andme	42,557	1,424,707	284
			C2_ALL_eur_leave_23andme	38,984	1,644,784	498
			C2_ALL_eur_leave_ukbb_23andme	32,494	1,316,207	271

b

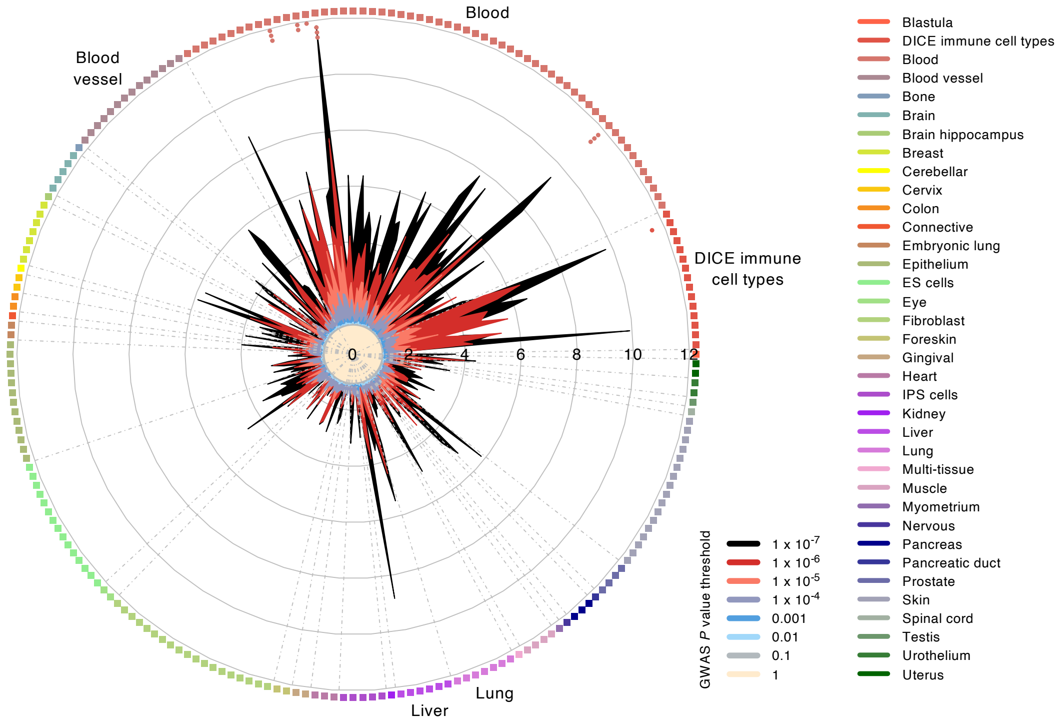

Supplementary Figure 1. COVID-19-risk associated GWAS SNPs and haploblocks.

(**a**) GWAS SNP datasets defined by the COVID-19 Host Genetics Initiative (see Methods), number of cases and controls in each study (release 5 from 18 January 2021), retrieved GWAS lead SNPs (GWAS association *P* value < 5 x 10^{-8}) and number of GWAS haploblocks. (**b**) The GARFIELD wheel plot displays functional enrichment of COVID-19-risk variants in chromatin accessibility sites identified by ATAC-seq analysis of immune cell types (DICE dataset) and cell types and tissues derived from ENCODE, GENCODE, and Roadmap Epigenomics ('peaks' dataset provided by GARFIELD). The radial lines show odds ratio (OR) values at each GWAS *P* value threshold (*T*) for each of the 224 samples tested, sorted by tissue cell types on the outer circle. Dots in the inner ring of the outer circle denote significant GARFIELD enrichment (if present) at $T < 1 \times 10^{-5}$ (outermost) to $T < 1 \times 10^{-8}$ (innermost) after multiple-testing correction for the number of effective annotations and are colored with respect to the cell type or tissue tested.

Supplementary Figure 2

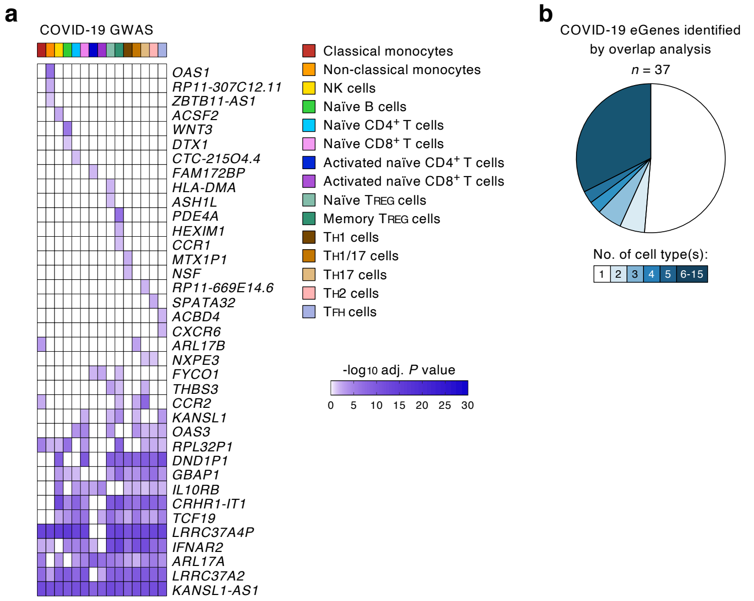

Supplementary Figure 2. COVID-19-risk associated GWAS eGenes identified by overlap with DICE eQTLs.

(**a**) Genes and cell types influenced by GWAS SNPs linked to COVID-19 severity and susceptibility (A, B, C). For each cell type (columns), the adj. association *P* value for the peak GWAS *cis*-eQTL associated with the indicated eGenes (rows) is shown. (**b**) Fractions of GWAS eGenes linked to COVID-19 severity and susceptibility (A, B, C) identified in varying numbers of cell types.

Supplementary Figure 3

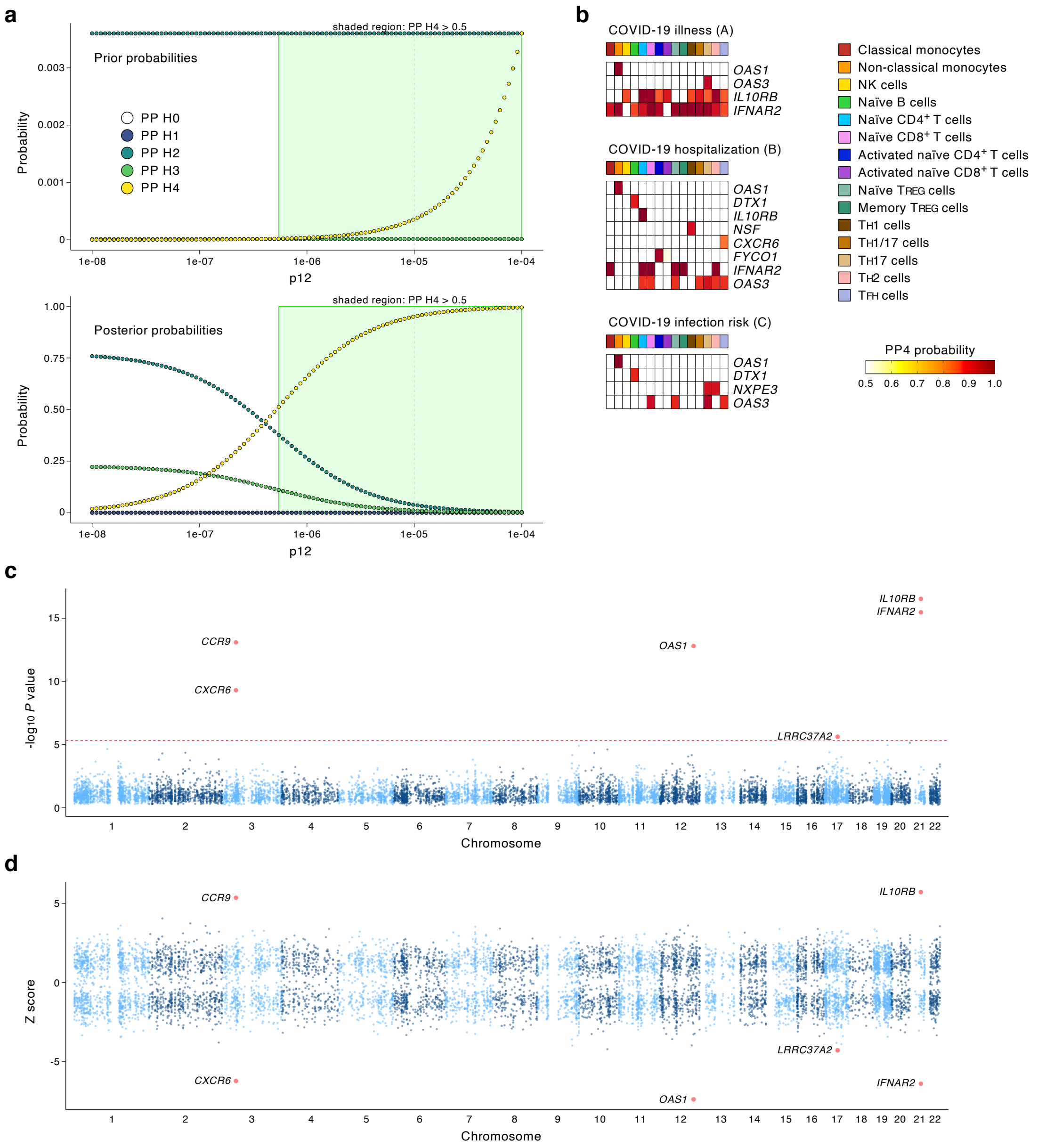

Supplementary Figure 3. COVID-19-risk associated GWAS eGenes identified by colocalization analysis and transcriptome-wide association studies.
(**a**) Sensitivity analysis of colocalization of COVID-19-risk GWAS variants and eQTLs for *IFNAR2* in naïve CD8+ T cells, as previously described[15]. The plot shows the prior and posterior probabilities as a function of p12 for the following hypothesis: PP H0 = variant with no association, PP H1= variant with association only with COVID-19 phenotype, PP H2 = variant with association only with gene expression, PP H3 = distinct causal variants, independent association of variants with COVID-19 phenotype and gene expression, PP H4 = shared causal variant, where a single causal SNPs is associated with both COVID-19 phenotype and gene expression. (**b**) Genes and immune cell types influenced by variants associated with three COVID-19 phenotypes: (A) critical COVID-19 illness, (B) moderate to severe COVID-19 illness requiring hospitalization and (C) reported SARS-CoV-2 infection (COVID-19 Host Genetics Initiative; release 5 from 18 January 2021; GWAS association *P* value $< 5 \times 10^{-8}$). For eGenes with colocalized signals (rows), the posterior probabilities of colocalized GWAS and eQTL signals (PP4) is shown for different immune cell types (columns). (**c**) Integrated TWAS (S-MulTiXcan[16]): gene-level Manhattan plot showing the association *P* value ($-\log_{10}$) for gene expression with COVID-19 phenotypes; results from integrated analysis across tissues is shown (see Methods). The red horizontal line shows gene-level Bonferroni corrected genome-wide significant *P* value threshold ($P < 4.81 \times 10^{-6}$). (**d**) Z scores showing the direction of effect for the genotype-inferred expression of transcripts that encode protein-coding genes in human immune cell types. Red circles indicate genes with Bonferroni corrected genome-wide significant *P* value threshold ($P < 4.81 \times 10^{-6}$).
