# Supplementary material for: COVID-19 genetic risk variants are associated with expression of multiple genes in diverse immune cell types
Source: Nat Commun. 2021 Nov 19;12:6760. doi: 10.1038/s41467-021-26888-3 (PMC8604964; doi:10.1038/s41467-021-26888-3)
Supplement: Supplementary file 3 — Description of Additional Supplementary Files [file 41467_2021_26888_MOESM3_ESM.pdf]

Description of Additional Supplementary Files

File Name: Supplementary Data 1
Description: Results from functional enrichment analysis (GARFIELD).

File Name: Supplementary Data 2
Description: List of eGenes in each immune cell type and activation condition, along with information on GWAS *cis*-eQTLs (GWAS association P value < 5×10^{-8}) that is associated with COVID-19 illness.

File Name: Supplementary Data 3
Description: Results from colocalization analysis.

File Name: Supplementary Data 4
Description: Results from single-tissue TWAS analysis (SPrediXcan).

File Name: Supplementary Data 5
Description: Results from integrated TWAS analysis (SMulTiXcan).

File Name: Supplementary Data 6
Description: Results from fine-mapping (FINEMAP).

File Name: Supplementary Data 7
Description: Transcription factor motifs perturbed by COVID19-risk associated variants.

File Name: Supplementary Data 8
Description: Details of sequencing libraries of H3K27ac HiChIP and ATAC-seq.
