# Supplementary material for: COVID-19 genetic risk variants are associated with expression of multiple genes in diverse immune cell types
Source: Nat Commun. 2021 Nov 19;12:6760. doi: 10.1038/s41467-021-26888-3 (PMC8604964; doi:10.1038/s41467-021-26888-3)
Supplement: Supplementary file 12 — Reporting Summary [file 41467_2021_26888_MOESM12_ESM.pdf]

nature portfolio

Corresponding author(s): Pandurangan Vijayanand

Last updated by author(s): September 17, 2021



# Reporting Summary

Nature Portfolio wishes to improve the reproducibility of the work that we publish. This form provides structure for consistency and transparency in reporting. For further information on Nature Portfolio policies, see our Editorial Policies and the Editorial Policy Checklist.

## Statistics

For all statistical analyses, confirm that the following items are present in the figure legend, table legend, main text, or Methods section.

| n/a | Confirmed | |
|---|---|---|
| ☐ | ☒ | The exact sample size (*n*) for each experimental group/condition, given as a discrete number and unit of measurement |
| ☐ | ☒ | A statement on whether measurements were taken from distinct samples or whether the same sample was measured repeatedly |
| ☐ | ☒ | The statistical test(s) used AND whether they are one- or two-sided<br>*Only common tests should be described solely by name; describe more complex techniques in the Methods section.* |
| ☐ | ☒ | A description of all covariates tested |
| ☐ | ☒ | A description of any assumptions or corrections, such as tests of normality and adjustment for multiple comparisons |
| ☐ | ☒ | A full description of the statistical parameters including central tendency (e.g. means) or other basic estimates (e.g. regression coefficient) AND variation (e.g. standard deviation) or associated estimates of uncertainty (e.g. confidence intervals) |
| ☐ | ☒ | For null hypothesis testing, the test statistic (e.g. *F*, *t*, *r*) with confidence intervals, effect sizes, degrees of freedom and *P* value noted<br>*Give P values as exact values whenever suitable.* |
| ☐ | ☒ | For Bayesian analysis, information on the choice of priors and Markov chain Monte Carlo settings |
| ☒ | ☐ | For hierarchical and complex designs, identification of the appropriate level for tests and full reporting of outcomes |
| ☐ | ☒ | Estimates of effect sizes (e.g. Cohen's *d*, Pearson's *r*), indicating how they were calculated |

*Our web collection on statistics for biologists contains articles on many of the points above.*

## Software and code

Policy information about availability of computer code

| | |
|---|---|
| Data collection | No code was used to collect data in the study. |
| Data analysis | GraphPad Prism v9.1.0 (https://www.graphpad.com/)<br>WashU Epigenome browser (https://epigenomegateway.wustl.edu/)<br>UCSC Genome Browser (https://genome.ucsc.edu/)<br>Custom scripts written in R v3.4.3 (https://www.r-project.org/)<br>FitHiChIP (https://github.com/ay-lab/FitHiChIP)<br>ChIPLine (https://github.com/ay-lab/ChIPLine)<br>Bowtie2 v2.3.3.1 (http://bowtie-bio.sourceforge.net/bowtie2/index.shtml)<br>MACS2 v2.1.0 (https://github.com/taoliu/MACS)<br>Picard (http://broadinstitute.github.io/picard)<br>SAMtools v1.6 (http://samtools.sourceforge.net/)<br>bedtools v2.26.0 (https://bedtools.readthedocs.io/en/latest/)<br>HLApers (https://github.com/genevol-usp/HLApers)<br>STAR (https://github.com/alexdobin/STAR)<br>Salmon (https://combine-lab.github.io/salmon/)<br>IMGTHLA database (https://github.com/ANHIG/IMGTHLA.git)<br>PLINK v1.90b3w (http://zzz.bwh.harvard.edu/plink/summary.shtml)<br>SNPTracker (http://grass.cgs.hku.hk/limx/snptracker/)<br>deepTools v2.0 (https://deeptools.readthedocs.io/en/develop/)<br>Matrix eQTL v2.2 (http://www.bios.unc.edu/research/genomic_software/Matrix_eQTL/)<br>COLOC v4.0.4 (https://github.com/chr1swallace/coloc)<br>PrediXcan (https://github.com/hakyimlab/PrediXcan) |

MetaXcan (https://github.com/hakyimlab/MetaXcan)
GARFIELD v2 (https://www.ebi.ac.uk/birney-srv/GARFIELD/)
FINEMAP (https://github.com/FINNGEN/finemapping-pipeline)
Colocalization analysis, TWAS analysis and GWAS overlap analysis (https://github.com/vijaybioinfo)
ATAC-seq and HiChIP data analysis (https://github.com/ay-lab)

For manuscripts utilizing custom algorithms or software that are central to the research but not yet described in published literature, software must be made available to editors and reviewers. We strongly encourage code deposition in a community repository (e.g. GitHub). See the Nature Portfolio guidelines for submitting code & software for further information.

## Data

Policy information about availability of data

All manuscripts must include a data availability statement. This statement should provide the following information, where applicable:

- Accession codes, unique identifiers, or web links for publicly available datasets
- A description of any restrictions on data availability
- For clinical datasets or third party data, please ensure that the statement adheres to our policy

The DICE project is providing anonymized data for public access at http://dice-database.org. Individual-specific RNA-sequencing and genotype data, H3K27ac ChIP-seq and HiChIP data in 5 common immune cell types has been previously reported (Schmiedel et al. Cell 2018, Chandra et al. Nature Genetics 2021). H3K27ac ChIP-seq and HiChIP data for non-classical monocytes and ATAC-seq data for 15 DICE cell types was newly generated. All datasets have been deposited in the Database of Genotypes and Phenotypes (dbGaP accession number: phs001703.v4.p1). All relevant data supporting the findings of this study are available from the corresponding author upon reasonable request.

# Field-specific reporting

Please select the one below that is the best fit for your research. If you are not sure, read the appropriate sections before making your selection.

☒ Life sciences ☐ Behavioural & social sciences ☐ Ecological, evolutionary & environmental sciences

For a reference copy of the document with all sections, see nature.com/documents/nr-reporting-summary-flat.pdf

# Life sciences study design

All studies must disclose on these points even when the disclosure is negative.

| | |
|---|---|
| Sample size | For the DICE project, a total of 91 healthy volunteers were recruited in the San Diego area and provided leukapheresis samples at the San Diego Blood Bank (SDBB) after written informed consent. Leukapheresis samples from study subjects have been collected and cryopreserved as part of the NIH funded grant (R24AI108564). |
| Data exclusions | No data was excluded. |
| Replication | We used DICE eQTL (Schmiedel et al. Cell 2018) and cis-interactome project (Chandra et al. Nature Genetics 2021) to show reproducibility. ChIP-seq and HiChIP were performed on six biological replicates. ATAC-seq was performed in technical duplicates for two study subjects. |
| Randomization | Randomization was not performed. The employed methods involve unbiased quantification, the data presented did not require randomization. |
| Blinding | Blinding was not performed. The employed methods involve unbiased quantification, the data presented did not require blinding. |

# Reporting for specific materials, systems and methods

We require information from authors about some types of materials, experimental systems and methods used in many studies. Here, indicate whether each material, system or method listed is relevant to your study. If you are not sure if a list item applies to your research, read the appropriate section before selecting a response.

## Materials & experimental systems

| n/a | Involved in the study | |
|---|---|---|
| ☐ | ☒ | Antibodies |
| ☒ | ☐ | Eukaryotic cell lines |
| ☒ | ☐ | Palaeontology and archaeology |
| ☒ | ☐ | Animals and other organisms |
| ☐ | ☒ | Human research participants |
| ☒ | ☐ | Clinical data |
| ☒ | ☐ | Dual use research of concern |

## Methods

| n/a | Involved in the study | |
|---|---|---|
| ☐ | ☒ | ChIP-seq |
| ☒ | ☐ | Flow cytometry |
| ☒ | ☐ | MRI-based neuroimaging |

## Antibodies

| | |
|---|---|
| Antibodies used | The premium H3K27ac polyclonal antibody from Diagenode (C15410196) was used for H3K27ac HiChIP and ChIP-seq, as described previously (Chandra et al. Nat Genet 2021). FACS antibodies and the underlying FACS gating strategy were described previously (Schmiedel et al. Cell 2018). |
| Validation | The antibodies have been validated by the respective manufacturer, tested on human cell lines and primary cells. |

## Human research participants

Policy information about studies involving human research participants

| | |
|---|---|
| Population characteristics | The DICE cohort consisted of 54 males and 37 female subjects with a median age of 27 years (range 18 to 61 years). All study subjects self-reported ethnicity and race details, and were tested negative for hepatitis B, hepatitis C and human immunodeficiency virus (HIV). Details of the study subjects were provided previously (Schmiedel et al. Cell 2018). |
| Recruitment | For the DICE project, a total of 91 healthy volunteers were recruited in the San Diego area and provided leukapheresis samples at the San Diego Blood Bank (SDBB) after written informed consent. |
| Ethics oversight | The Institutional Review Board (IRB) of the La Jolla Institute for Immunology (LJI) approved the study (IRB protocol no. SGE-121-0714). |

Note that full information on the approval of the study protocol must also be provided in the manuscript.

## ChIP-seq

### Data deposition

☒ Confirm that both raw and final processed data have been deposited in a public database such as GEO.

☒ Confirm that you have deposited or provided access to graph files (e.g. BED files) for the called peaks.

| | |
|---|---|
| Data access links<br>*May remain private before publication.* | Individual-specific RNA-sequencing and genotype data, HiChIP and ChIP-seq data and ATAC-seq data are available from the database of Genotypes and Phenotypes (dbGaP accession number: phs001703.v4.p1). |
| Files in database submission | Fastq |
| Genome browser session<br>(e.g. UCSC) | WashU browser session IDs containing HiChIP loop calls and aggregated ChIP-seq tracks for 6 cell types: a3673fc0-9815-11eb-ab74-c9be432610d8. The user needs to visit https://epigenomegateway.wustl.edu/, provide the session ID, click "retrieve", select "restore" to visualize the tracks. |

### Methodology

| | |
|---|---|
| Replicates | Six biological replicates were used to provide sufficient confidence to conclusions derived from H3K27ac ChIP-seq on immune cell types, as described previously (Chandra et al. Nat Genet 2021). |
| Sequencing depth | Libraries were sequenced on an Illumina NovaSeq6000 sequencer to obtain 100-bp paired-end reads. |
| Antibodies | The premium anti-human H3K27ac polyclonal antibody from Diagenode (C15410196) was used for H3K27ac HiChIP and ChIP-seq (Lot No. A1723-0041D). |
| Peak calling parameters | ChIP-seq reads were aligned using Bowtie2 to hg19 reference genome, using the parameters -k 4 --mm --threads 8 -X 2000. Uniquely mapped reads with MAPQ >= 30 were retained, and duplicate reads were discarded using Picard (http://broadinstitute.github.io/picard). For each cell type, the resulting de-duplicated aligned reads (in .bam format) were merged using samtools (http://samtools.sourceforge.net/) for all six donors to produce aggregate ChIP-seq reads for each cell type. These merged alignment files were then applied to MACS2 (version 2.1.1) for peak calling, using the parameters -f BAM -g 'hs' --nomodel --extsize 147 --keep-dup 1 -q 0.01. |
| Data quality | Number of peaks with FDR 0.05 in Non-classical monocytes (peaks from aggregated ChIP-seq reads): 91,403 |
| Software | ChIP-seq data was processed as described above, using the custom pipeline ChIPLine developed in our lab (https://github.com/ay-lab/ChIPLine). |


March 2021
